# What can management theories offer evidence-based practice? A comparative analysis of measurement tools for organisational context

**DOI:** 10.1186/1748-5908-4-28

**Published:** 2009-05-19

**Authors:** Beverley French, Lois H Thomas, Paula Baker, Christopher R Burton, Lindsay Pennington, Hazel Roddam

**Affiliations:** 1 School of Nursing and Caring Sciences, University of Central Lancashire, Preston, Lancashire, England, PR1 2HE, UK; 2 Pennine Acute Hospitals NHS Trust, North Manchester General Hospital, Manchester, England, M8 5RB, UK; 3 Centre for Health-Related Research, School of Healthcare Sciences, College of Health and Behavioural Sciences, Bangor University, Gwynedd, Wales, LL57 2EF, UK; 4 School of Clinical Medical Sciences (Child Health), University of Newcastle, Sir James Spence Institute, Royal Victoria Infirmary, Queen Victoria Road, Newcastle upon Tyne, England, NE1 4LP, UK; 5 School of Public Health and Clinical Sciences, University of Central Lancashire, Preston, Lancashire, England, PR1 2HE, UK

## Abstract

**Background:**

Given the current emphasis on networks as vehicles for innovation and change in health service delivery, the ability to conceptualise and measure organisational enablers for the social construction of knowledge merits attention. This study aimed to develop a composite tool to measure the organisational context for evidence-based practice (EBP) in healthcare.

**Methods:**

A structured search of the major healthcare and management databases for measurement tools from four domains: research utilisation (RU), research activity (RA), knowledge management (KM), and organisational learning (OL). Included studies were reports of the development or use of measurement tools that included organisational factors. Tools were appraised for face and content validity, plus development and testing methods. Measurement tool items were extracted, merged across the four domains, and categorised within a constructed framework describing the absorptive and receptive capacities of organisations.

**Results:**

Thirty measurement tools were identified and appraised. Eighteen tools from the four domains were selected for item extraction and analysis. The constructed framework consists of seven categories relating to three core organisational attributes of vision, leadership, and a learning culture, and four stages of knowledge need, acquisition of new knowledge, knowledge sharing, and knowledge use. Measurement tools from RA or RU domains had more items relating to the categories of leadership, and acquisition of new knowledge; while tools from KM or learning organisation domains had more items relating to vision, learning culture, knowledge need, and knowledge sharing. There was equal emphasis on knowledge use in the different domains.

**Conclusion:**

If the translation of evidence into knowledge is viewed as socially mediated, tools to measure the organisational context of EBP in healthcare could be enhanced by consideration of related concepts from the organisational and management sciences. Comparison of measurement tools across domains suggests that there is scope within EBP for supplementing the current emphasis on human and technical resources to support information uptake and use by individuals. Consideration of measurement tools from the fields of KM and OL shows more content related to social mechanisms to facilitate knowledge recognition, translation, and transfer between individuals and groups.

## Background

The context of managing the knowledge base for healthcare is complex. Healthcare organizations are composed of multi-level and multi-site interlacing networks that, despite central command and control structures, have strong front-line local micro-systems involved in interpreting policy direction [1]. The nature of healthcare knowledge is characterized by proliferation of information, fragmentation, distribution, and high context dependency. Healthcare practice requires coordinated action in uncertain, rapidly changing situations, with the potential for high failure costs [2]. The public sector context includes the influence of externally imposed performance targets and multiple stakeholder influences and values, the imperative to share good practice across organisational boundaries, and a complex and diverse set of boundaries and networks [3]. Having strong mechanisms and processes for transferring information, developing shared meanings, and the political negotiation of action [4,5] are therefore crucially important in public sector/healthcare settings, but it is not surprising that there are reports of problems in the organizational capacity of the public sector to effectively manage best practice innovation [6-11], particularly around issues of power and politics between different professional groups [12-17].

The development of capacity to implement evidence-based innovations is a central concept in UK government programmes in healthcare [18]. Strategies to improve evidence-based decision making in healthcare have only recently shifted emphasis away from innovation as a linear and technical process dominated by psychological and cognitive theories of individual behaviour change [19], toward organisational level interventions [20], with attention shifting toward the development of inter-organisational clinical, learning, and research networks for sharing knowledge and innovation [21-23], and attempts to improve capacity for innovation within the public sector [24].

Organisational capacity refers to the organisation's ability to take effective action, in this context for the purpose of continually renewing and improving its healthcare practices. Absorptive and receptive capacities are theorized as important antecedents to innovation in healthcare [25]. Broadly, the concept of absorptive capacity is the organization's ability to recognise the value of new external knowledge and to assimilate it, while receptive capacity is the ability to facilitate the transfer and use of new knowledge [26-31]. Empirical studies have identified some general antecedent conditions [32-34], and have tested application of the concept of absorptive capacity to healthcare [35,36], although receptive capacities are less well studied. Empirically supported features of organisational context that impact on absorptive and receptive capacities in healthcare include processes for identifying, interpreting, and sharing new knowledge; a learning organisation culture; network structures; strong leadership, vision, and management; and supportive technologies [25].

Public sector benchmarking is widely promoted as a tool for enhancing organisational capacity via a process of collaborative learning [37]. Benchmarking requires the collation and construction of best practice indicators for institutional audit and comparison. Tools are available to measure the organizational context for evidence-based healthcare practice [38-41], and components of evidence-based practice (EBP) including implementation of organisational change [42-45], research utilization (RU) [46], or research activity (RA) [47]. While organisational learning (OL) and knowledge management (KM) frameworks are increasingly being claimed in empirical studies in healthcare [48-53], current approaches to assessing organisational capacity are more likely to be underpinned by diffusion of innovation or change management frameworks [54].

Nicolini and colleagues [2] draw attention to the similarity between the KM literature and the discourse on supporting knowledge translation and transfer in healthcare [55-57], as well as between concepts of OL and the emphasis on collective reflection on practice in the UK National Health Service [58,59], but suggest that 'ecological segregation' between these disciplines and literatures means that cross-fertilisation has not occurred to any great extent. OL and KM literatures could be fruitful sources for improving our understanding of dimensions of organizational absorptive and receptive capacity in healthcare. We therefore aimed to support the development of a metric to audit the organizational conditions for effective evidence-based change by consulting the wider OL and knowledge literatures, where the development of metrics is also identified as a major research priority [60], including the use of existing tools in healthcare [2].

Definitions of KM vary, but many include the core processes of creation or development of knowledge, its movement, transfer, or flow through the organisation, and its application or use for performance improvement or innovation [61]. Early models of KM focused on the measurement of knowledge assets and intellectual capital, with later models focusing on processes of managing knowledge in organisations, split into models where technical-rationality and information technology solutions were central and academic models focusing on human factors and transactional processes [62]. The more emergent view is of the organisation as 'milieu' or community of practice, where the focus on explanatory variables shifts away from technology towards the level of interactions between individuals, and the potential for collective learning. However, technical models and solutions are also still quite dominant in healthcare [63].

Easterby-Smith and Lyles [64] consider KM to focus on the content of the knowledge that an organisation acquires, creates, processes, and uses, and OL to focus on the process of learning from new knowledge. Nutley, Davies and Walker [54] define OL as the way organisations build and organise knowledge and routines and use the broad skills of their workforce to improve organisational performance. Early models of OL focused on cognitive-behavioural processes of learning at individual, group, and organisational levels [65-67], and the movement of information in social or activity systems [68]. More recent practice-based theories see knowledge as embedded in culture, practice, and process, conceptualising knowing and learning as dynamic, emergent social accomplishment [69-72]. Organisational knowledge is also seen as fragmented into specialised and localised communities of practice, 'distributed knowledge systems' [73], or networks with different interpretive frameworks [74], where competing conceptions of what constitutes legitimate knowledge can occur [75], making knowledge sharing across professional and organization boundaries problematic.

While the two perspectives of KM and OL have very different origins, Scarbrough and Swan [76] suggest that differences are mainly due to disciplinary homes and source perspectives, rather than conceptual distinctiveness. More recently, there have been calls for cognitive and practice-based theories to be integrated in explanatory theories of how practices are constituted, and the practicalities of how socially shared knowledge operates [77,78]. Similarly, there have been calls for integrative conceptual frameworks for OL and knowledge [79,80], with learning increasingly defined in terms of knowledge processes [81,82].

Practice models have their limitations, particularly in relation to weaknesses in explaining how knowledge is contested and legitimated [83]. In a policy context that requires clinical decisions to be based on proof from externally generated research evidence, a comprehensive model for healthcare KM would need to reflect the importance of processes to verify and legitimate knowledge. Research knowledge then needs to be integrated with knowledge achieved from shared interpretation and meaning within the specific social, political, and cultural context of practice, and with the personal values-based knowledge of both the individual professional and the patient [84]. Much public sector innovation also originates from practice and practitioners, as well as external scientific knowledge [85,86]. New understandings generated from practice then require re-externalising into explicit and shared formal statements and procedures, so that actions can be defended in a public system of accountability.

Our own preference is for a perspective where multiple forms of knowledge are recognised, and where emphasis is placed on processes of validating and warranting knowledge claims. Attention needs also be directed towards the interrelationship between organisational structures of knowledge governance, such as leadership, incentive and reward structures, or the allocation of authority and decision rights, and the conditions for individual agency [87-89]. Our own focus is therefore on identifying the organizational conditions that are perceived to support or hinder organizational absorptive or receptive capacities, as a basis for practical action by individuals.

The indicators for supportive organisational conditions are to be developed by extracting items from existing tools, as in previous tools developed to measure OL capability [90]. Existing tools are used because indicators are already empirically supported, operationalised, and easily identified and compared, and because our primary focus is one of utility for practice [91], by specifying 'the different behavioural and organisational conditions under which knowledge can be managed effectively' [92p ix]. Measurement tools that were based on reviews of the literature in the respective fields of KM and learning organisations were chosen as comparison sources to assess the comprehensiveness of the current tools in healthcare, and to improve the delineation of the social and human aspects of EBP in healthcare. If this preliminary stage proves fruitful in highlighting the utility of widening the pool for benchmark items, future work aims to compare the source literatures for confirming empirical evidence, with further work to test the validity and reliability of the benchmark items.

## Methods

A structured literature review was undertaken to collate measurement tools for organisational context from the domains of research use or RA in healthcare, or for KM or OL in the management or organisational science literature.

### Search and screening

A search of electronic databases from inception to March 2006 was carried out on MEDLINE, CINAHL, AMED, ZETOC, IBSS, Web of Science, National Research Register, Ingenta, Business Source Premier, and Emerald. Measurement tools were included if they were designed to measure contextual features of whole organisations, or sub-units such as teams or departments. Tools needed to include at least one item relating to organisational factors influencing RU, RA, KM, or OL. To be included, papers had to report a structured method of tool development and psychometric testing.

### Data extraction and analysis

Individual reviewers (BF, PB, LT) extracted items relating to organisational context from each measurement tool. Items were excluded if they focused solely on structural organisational factors not amenable to change (*e.g.*, organisational design, size; inter-organisational factors) and environment (*e.g.*, political directives); or characteristics of the commercial context that were not applicable in a public service context. Some tools had items expressed as staff competencies (*e.g.*, 'Staff in our organization have critical appraisal skills...') or organisational processes (*e.g.*, 'Our organization has arrangements with external expertise...' [93]). Items such as these were included and interpreted in terms of the availability of an organisational resource (*e.g.*, facilities for learning critical appraisal skills, or availability of external expertise). However, some items were not expressed in a way that could be inferred as an organisational characteristic (*e.g.*, 'Our employees resist changing to new ways of doing things' [94]), and were excluded.

### Category analysis

Initially, similar items from different measurement tools were grouped together, *e.g.*, 'I often have the opportunity to talk to other staff about successful programmes...' [95] and 'employees have the chance to talk among themselves about new ideas...' [96]. After an initial failed attempt to categorize all items using an existing diffusion of innovation framework [25], the review team constructed categories of organisational attributes by grouping items from across all the measurement instruments, and refining, expanding, or collapsing the groupings until a fit was achieved for all extracted items. The material is illustrated in Table 1 by items allocated to two attributes: involving the individual, and shared vision/goals (tool source in brackets – see Table 2[97-104]). While broadly similar, it can be seen that items from the different domains are expressed differently, and there was some judgement involved in determining the similarity of meaning across domains. It can also be seen that for some categories, particular domains of tool did not contribute any items, while other domains contributed multiple items.

**Table 1:** Example of categorisation of items extracted from measurement tools

Research activity	Research utilisation	Knowledge management	Organisational learning
Involving the individual			

Organisation ensures staff involvement in discussion on how research evidence relates to organisational goals (KEYS)[93] Expectation from organisation for staff involvement (ABC)[107]			Managers in this organisation frequently involve employees in important decisions (OLS2)[95] Part of this firms' culture is that employees can express their opinions and make suggestions regarding the procedures and methods in place for carrying out tasks (OLC2)[96]

Shared vision/goals			

What I do links with the Directorate's plans (ABC)[107] The development work of individuals links with the Directorate's plans (RandD)[47]		I usually agree with the direction set by this organisation's leadership (KMS)[97]	Senior managers and employees share a common vision of what our work should accomplish (OLS2)[95]

**Table 2:** Measurement tools included for item extraction

Number	Short name	Research activity
1	ABC	ABC Survey [107]

2	BARR	BARRIERS Scale [46]

3	BART	Barriers and Attitudes to Research in Therapies [98]

4	KEYS	KEYS – Knowledge Exchange Yields Success Questionnaire [93]

5	NDF	Nursing Department Form [106]

		Research utilization

6	RUS	RU Scale [99,100]

7	RUSI	RU Survey Instrument [105,108]

8	RUIN	Research Use in Nursing Practice Instrument [101]

9	RandD	R and D Culture Index [47]

		Knowledge Management

10	CCS	Collaborative Climate Survey [102]

11	KMAT	KM Assessment Tool [103]

12	KMQ	KM Questionnaire [109]

13	KMS	KM Scan [97]

		Organisational Learning

14	OLC1	OL Capacity [104]

15	OLC2	OL Capability Scale [96]

16	OLC3	OL Construct [94]

17	OLS1	OL Scale [110]

18	OLS2	OL Survey [95]

We conducted three rounds of agreement with the fit of items to categories: an initial round using categories derived from the diffusion of innovation framework by Greenhalgh and colleagues [25], which was rejected because of the lack of fit for numerous items; a second round with our own constructed categorization framework built from grouping items; and a third and final round for reviewers to check back that all items from their measurement tools had been included and adequately categorized in the constructed framework. Between each round, joint discussions were held to agree refinements to categories and discuss any disagreement. Using this process, agreement was reached between all reviewers on the inclusion and categorization of all items. An independent reviewer (LP) then checked validity of extraction, categorization, and merging by tracing each composite attribute back to the original tool, agreeing its categorization, then reviewing each tool to ensure that all relevant items were incorporated. Items queried were re-checked.

## Results

Thirty tools were identified and appraised [see Additional file 1]. Based on the inclusion criteria for tool development and testing, 18 tools with 649 items in total were selected. These are listed in Table 2, with information on development and psychometric testing [see Additional File 2] The number of the tool from Table 2 will be used in subsequent tables.

In total, 261 items related to organisational context were extracted from the measurement tools. For two tools [105,106], the full text of each item was not available, so the names of the categories of measurement for which results were reported were used as items, *e.g.*, organisational climate for change.

### Final model

**Figure 1:**
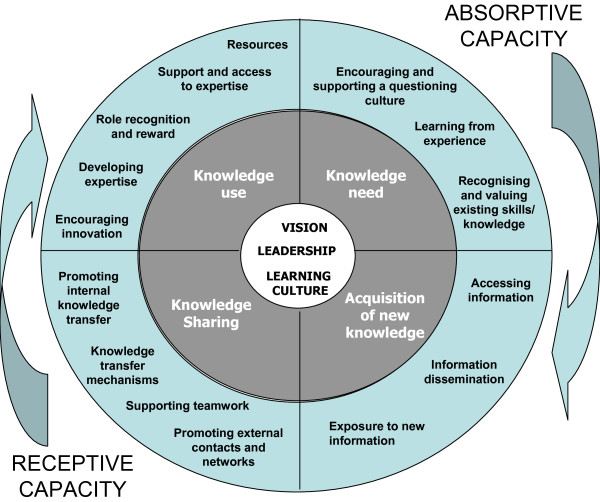
**Model of categories and organisational attributes**.

Figure 1 illustrates the final category structure constructed to account for all of the items from the measurement tools. Seven broad categories gave a best fit for the items. The central white circle of the diagram shows three core categories of vision, leadership, and a learning culture. The middle ring shows four categories of activity: 'knowledge need and capture' and 'acquisition of new knowledge' (relating to organisational absorptive capacity); and 'knowledge sharing' and 'knowledge use' (related to organisational receptive capacity). The outer ring illustrates the organisational attributes contributing to each category.

### Tool item analysis

Table 3 summarises the organisational attributes for each category. Attributes are based on a composite of items extracted from the tools across the four domains. An example of a single tool item is given to illustrate the source material for each attribute.

**Table 3:** Details of attributes in each category, and example of tool items

Category	Attribute	Examples of individual tool items + source
OL culture	Climate:, *e.g.*, openness, respect, trust	Open communication is a characteristic of the Department (CCS)[102]
	
	Learning as a key value	The basic values of the Department include learning as a key to improvement (OLC3)[94]
	
	Involving the individual	Managers frequently involve staff in important decisions (OLS2)[95]
	
	Valuing the individual	The organisation considers individuals to be an asset (OLS1)[110]

Vision	Existence of key strategic aims	Managing knowledge is central to the organisation's strategy (KMAT)[103]
	
	Existence of policies and infrastructures	There are specific infrastructures to support the research process (ABC)[107]
	
	Communication	Management clearly communicates key research strategy and priorities (BART)[98]
	
	Shared vision/goals	There is widespread support and acceptance of the organisation's mission statement (OLS2)[95]

Leadership	Presence of leadership	Strong professional leadership (KEYS)[93]
	
	Existence of committees and representation	Nursing representation on research committee, council etc (ABC)[107]
	
	Managerial processes and attributes	Management proactively addresses problems (OLC1)[104]

Knowledge need	Existence of a questioning culture	Nurses are encouraged to question their practices (ABC)[107]
	
	Learning from experience	Problems are discussed openly and without blame (OLS1)[110]
	
	Recognising and valuing existing knowledge	There are best practice repositories in my organisation (KMQ)[109]

Acquisition of new knowledge	Accessing information	Network access to information databases available to all (OLS1)[110]
	
	Information dissemination	Use of communication skills to present information in a 'user friendly' way (BART)[98]
	
	Exposure to new information	Attendance at conferences/presentations that give information (RUS)[99,100]

Knowledge sharing	Promoting internal knowledge transfer	Employees are encouraged to discuss xperiences/expertise with colleagues (KMS)[97]
	
	Supporting teamwork	Multi-professional review and audit (ABC)[107]
	
	Knowledge transfer technology/mechanisms	Technology to support collaboration is available and placed rapidly in the hands of employees (KMAT)[103]
	
	Promoting external contacts	We have a system that allows us to learn successful practices from other organisations (OLS2)[95]

Knowledge use	Encouraging innovation	This firm promotes experimentation and innovation as a way of improving the work processes (OLC2).[96]
	
	Developing expertise	We are encouraged to attend training programmes (KMQ)[109]
	
	Role recognition and incentives/reward	Nurses who participate in the research process receive recognition for their involvement (ABC)[107]
	
	Support and access to expertise a) internal-management b) internal – peers c) internal – others b) external	Cooperative agreements with Universities etc formed (KMS)[97]
	
	Access to resources a) funding b) time c) evaluation and data capture technology d) authority	My organisation provides resources for the utilisation of nursing research (RandD)[47]

The marked areas in Table 4 identify the measurement tool source of each organisational attribute. The percentages are derived from the number of times an item is included in a category, compared with the total possible in each domain, *e.g.*, there were two items from RA tools included in the learning culture category, out of a possible total of 16 items. The results for each category are discussed below:

**Table 4:** Categorisation of measurement tool items

Domain:	Research activity (RA 1–4)				Research utilisation (RU 5–9)					Knowledge management (KM 10–13)				Organisational Learning (OL 14–18)				
*Tool:	1	2	3	4	5	6	7	8	9	10	11	12	13	14	15	16	17	18

Learning culture																		

Climate										x	x	x						

Learning as a key value											x				x	x		

Involving the individual	x		x						x						x			x

Valuing the individual									x	x		x					x	x

% coverage	12%				5%					37%				30%				

Vision																		

Key strategic aim	x		x						x		x					x		

Policies and infrastructures	x				x												x	

Communication	x		x									x			x	x	x	x

Shared vision/goals			x	x						x			x		x	x	x	x

% coverage	44%				10%					25%				50%				

Leadership																		

Leadership			x	x									x					

Committees/representation	x				x		x			x								

Managerial attributes			x				x							x				x

% coverage	33%				12%					17%				13%				

Knowledge need																		

Questioning culture	x			x			x				x							x

Learn from experience				x								x	x		x	x	x	x

Existing knowledge									x		x	x		x	x		x	

	17%				13%					42%				53%				

Acquiring new knowledge																		

Accessing information	x	x	x		x	x	x	x	x								x	

Information dissemination			x			x						x		x			x	

Exposure: new information	x	x			x			x	x				x				x	

% coverage	50%				60%					17%				27%				

Knowledge sharing																		

Internal knowledge transfer	x		x			x		x		x	x	x	x	x	x	x	x	x

Supporting teamwork		x		x											x	x	x	

Transfer technology											x	x		x		x	x	

External contacts			x									x	x	x	x		x	x

% coverage	31%				10%					50%				75%				

Knowledge use																		

Encouraging innovation	x		x			x	x		x	X	x	x	x	x	x	x	x	x

Developing expertise	x	x	x	x			x		x			x	x	x			x	x

Role recognition/reward	x	x	x	x	x	x	x		x		x		x		x	x		x

Access to expertise	x	x	x	x	x	x	x	x				x	x	x	x		x	

Access to resources	x	x	x	x		x			x		x	x	x	x			x	

% coverage	90%				60%					65%				64%				

*See Table 2 for full names and references for measurement tools

### Learning culture

OL and KM tools were the most frequent source of these attributes, with seven out of nine tools covering attributes in this category, although none of the tools covered all of the attributes. Three RA/RU tools covered the attribute of 'involving the individual', with one of the RU tools also including the attribute of 'valuing the individual'. Each attribute was sourced from between three and five tools across all domains. The most representation was sourced from KM tools.

### Vision

Eight out of nine of the OL/KM tools, and five out of nine RA/RU tools included attributes from this category. The most common attribute was 'shared vision/goals' (eight tools), and the least common was 'policies and infrastructures' (three tools). The most representation was sourced from OL tools.

### Leadership

All of the domains included some reference to attributes of management or leadership. Five out of nine RA/RU tools and four out of nine KM/OL tools included items related to leadership. The most representation was in RA tools.

### Knowledge need

All of the OL tools and three out of four of the KM tools included items related to attributes of this category. They were less commonly sourced from RA and RU tools. The most common attribute was 'learning from experience' (seven tools). The most representation was sourced from OL tools.

### Acquiring new knowledge

Attributes in this category were more commonly sourced in RA/RU tools. Attributes were sourced from between five and nine tools out of the total of 18 tools across all domains, and each attribute was covered in each domain, except 'accessing information', which was not covered in any KM tool. The most representation was sourced from RU tools.

### Knowledge sharing

Most OL/KM tools included multiple attributes from this category, all RA tools included one or two items, but only two out of five RU tools included one attribute. 'Promoting internal knowledge transfer' was the most common attribute, included in 13 out of 18 tools, with 'promoting external contacts' included in seven tools. The other items were included in five tools. The most representation for this category was sourced from OL tools.

### Knowledge use

Overall, this was the largest and most populated category. The most common attributes referred to were 'encouraging innovation', included in 14 out of 18 tools, and 'role recognition/reward', referred to in 13 tools. Each of the other attributes was also referred to in at least eight tools. All attributes were sourced from all domains. The most representation for this category was sourced from RA tools.

### Analysis of tool coverage

Table 4 also summarises how well each tool domain covers the constructed categories and attributes. The results for each domain are discussed below:

### RA tools

The category with the most representation in the RA tools was 'knowledge use', with items in the category of 'acquiring new knowledge' and 'vision' also well represented. The categories of 'knowledge need' and 'knowledge sharing' were less well reflected across the RA tools. Two attributes of 'recognising and valuing existing knowledge' and 'knowledge transfer technology' did not appear in any RA tool. Five attributes appeared in only one of the tools. Four attributes of 'developing expertise, role recognition and reward','support/access to expertise', and'access to resources' were common to all tools. Two tools had relatively good coverage of the attributes: the ABC survey [107], with 14 out of 26 attributes covered, and the KEYS Questionnaire [93] with 15 out of 26 attributes covered.

### RU tools

This was the domain with the least coverage overall, commonly centered in the categories of 'acquiring new knowledge' and 'knowledge use'. The other categories were poorly represented. The attribute of 'accessing information' was common to all tools, with 'role recognition/reward', and 'support/access to expertise' common to four out of five tools. The tool which covered the most attributes (10 out of 26) was the RU Survey Instrument [105,108].

### KM tools

The KM tools covered all of the categories, with more common representation in the categories of 'learning culture', 'knowledge need', 'knowledge sharing' and 'knowledge use', but individual tools varied in their emphasis. The categories of 'leadership' and 'acquisition of new knowledge' were the least well represented. Two attributes were included in all four tools: 'promoting internal knowledge transfer', and 'encouraging innovation'.'Learning climate' and 'access to resources' were included in three out of four tools. Five attributes were not represented in any tool: 'involving the individual','policies and infrastructures','managerial attributes','accessing information', and 'supporting teamwork'. The tool with the best overall coverage of the attributes (13 out of 26) was the KM Questionnaire [109].

### OL tools

OL tools covered all categories, and generally had more consistent coverage than other domains of the categories 'vision', 'knowledge need' and 'knowledge sharing'. Single attributes relating to 'promoting internal knowledge transfer', and 'encouraging innovation' were covered in all five tools, with the attributes of 'communication', 'shared vision and goals','learning from experience', and 'promoting external contacts/networks' covered in four out of five tools. 'Key strategic aims','policies and infrastructures','questioning culture', 'accessing information', and 'exposure to new information' were only covered in one out of the five tools. The OL Scale [110] covered 17 out of the 26 possible attributes. The other four tools covered between 8 and 11 attributes.

### Comparison of support for benchmark items: what can EBP tools learn from the KM and OL literature?

**Figure 2:**
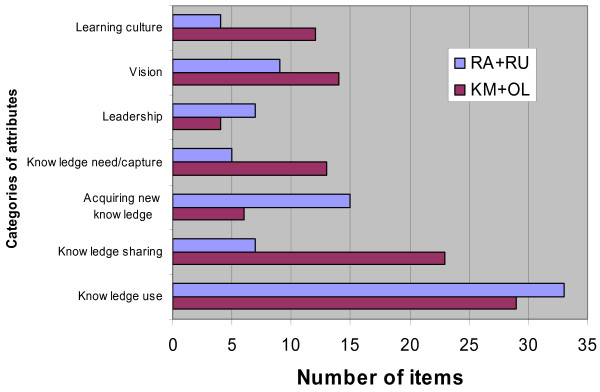
Comparison of RA/RU versus KM/OL measurement tools.

While each of the composite attributes is supported by items extracted from at least three measurement tools, there are differences in emphasis across the domains. To consider the potential contribution of the newer domains of KM/OL, the number of items from these domains have been pooled and compared against the number of items sourced from the domains commonly represented in the healthcare literature, *i.e.*, RA/RU. Figure 2 illustrates that the KM and OL literature focus more on 'learning culture', 'vision', 'knowledge need', and 'knowledge sharing'. The RA and RU literatures have a stronger emphasis on 'leadership', 'acquiring new knowledge', and 'knowledge use'.

## Discussion

The importance of understanding context has been reiterated by the High Level Clinical Effectiveness group [18]. This project was developed in response to perceived limitations in the conceptualisation and measurement of organisational context for EBP in healthcare. We wanted to move away from the rather narrow focus on RU and change management to include wider process and practice-based perspectives from the KM and OL literature. Our analysis of existing measurement tools has confirmed differences in emphasis across the domains. Measurement tools for RA and RU focus more on access to new information, leadership, and resources for change, and less on recognizing, valuing, and building shared knowledge. This is congruent with the culture of 'rationality, verticality, and control' [[6] p660] in healthcare, but the lack of attention to social context may be one reason why attempts to improve practice by influencing the behaviour of individual practitioners have variable results [111].

The emphasis in KM and OL tools on shared vision, learning culture, and sharing existing knowledge reflects a more socially mediated view of knowledge. If it is groups and networks that generate the meaning and value to be attached to evidence, organisational efforts to improve EBP would need to do more to shift towards supporting horizontal knowledge transfer. Networks have emerged as a recent UK government strategy for moving health research into action by creating clusters that break down disciplinary, sectoral, and geographic boundaries, but communication structures alone are unlikely to be successful for knowledge transfer across specialized domains [4,112] without additional mechanisms to support the transfer of practice and process knowledge [73].

Since this search was conducted, three additional tools to measure organisational context in quality improvement related areas have been reported [113-115]. Each of these tools has strengths, including attributes such as feedback that are not included in our model, but none have comprehensive coverage of all of the attributes identified in this study.

A potential weakness in using existing tools as sources in this study is that they might not reflect the latest theories and concepts, because tool development tends to lag behind conceptual development. This might result in inadvertent bias towards earlier more technical models, and we acknowledge that the existing tools do largely adopt a structuralist perspective. While the items contained in the existing measurement tools can only ever provide a rather simplistic reflection of complex phenomena, we felt that including them was better than not attempting to express them at all. However, while new perspectives are worth investigating, the unquestioning interdisciplinary transfer of theory also needs care. Compared with the public sector, there are differences in the types of problems, the availability of information and resources, and the motivations for evidence uptake and use. KM theory supposes an identified knowledge need, scarce information, and a workforce motivated by external incentive in a resource-rich environment. EBP on the other hand requires compliance with externally produced information for predominantly intrinsic reward, with high innovation costs in a resource-limited environment. A number of studies have identified some of the difficulties of knowledge sharing in the public sector [1,6,11,16,48,116,117]. Organisational theory may not transfer well into healthcare if EBP is viewed as a process of social and political control to promote compliance with centrally derived policy, rather than a generative process to make best use of available knowledge.

## Conclusion

Assessing organisational absorptive and receptive capacity with the aim of improving organizational conditions is postulated as a first step in supporting a research informed decision-making culture. Foss [87] suggests the emergence of a new approach referred to as knowledge governance: the management of the mechanisms that mediate between the micro-processes of individual knowledge and the outcomes of organisational performance. But what would this mean in practice? The kinds of support which KM and OL tools include as standard, but that are not well reflected in existing tools to measure context in healthcare, would include effort to detect and support emergent and existing communities of practice; encourage and reward individuals and groups to ask questions; discuss and share ideas across knowledge communities; and support the progression, testing, and adopting of new ideas by embedding them in systems and processes.

The processes by which individual- and group-level knowledge are collated into organisational level capability to improve care are less clear. If social networks of individuals are to be facilitated to undertake repeated, ongoing, and routine uptake of evidence within their daily practice, we also need to extend our thinking even further toward considering the organisational contextual features that would support the collective sense-making processes of key knowledge workers.

## Competing interests

The authors declare that they have no competing interests.

## Authors' contributions

BF, LHT and PB undertook searching, data extraction and categorisation. LP undertook external auditing of data analysis. BF drafted the final paper. CRB, LP and HR contributed to the conceptual design of the overall project, and acted as critical readers for this paper. All authors approved the final version of the manuscript.

## Supplementary Material

Additional file 1 **Measurement tools identified by the search**. Titles and bibliographic reference for all measurement tools identified as potentially relevant. Click here for file

Additional file 2 **Details of development and psychometric testing of included measurement tools**. Provides background information on psychometric properties of included measurement tools. Click here for file

## References

[B1] Pope C, Robert G, Bate P, le May A, Gabbay J (2006). Lost in translation: a multi-level case study of the metamorphosis of meanings and action in public sector organizational innovation. Public Admin.

[B2] Nicolini D (2008). Managing knowledge in the healthcare sector: a review. Int J Manag Rev.

[B3] Rashman L, Withers E, Hartley J (2008). Organizational Learning, Knowledge and Capacity: a systematic literature review for policy-makers, managers and academics.

[B4] Carlile PR (2004). Transferring, translating, and transforming: An integrative framework for managing knowledge across boundaries. Organ Sci.

[B5] Carlile PR (2002). A pragmatic view of knowledge and boundaries: boundary objects in new product development. Organ Sci.

[B6] Bate SP, Robert G (2002). Knowledge management and communities of practice in the private sector: Lessons for modernizing the national health service in England and Wales. Public Admin.

[B7] Newell S, Edelman L, Scarborough H, Swan J, Bresen M (2003). 'Best practice' development and transfer in the NHS: the importance of process as well as product knowledge. Health Serv Manage Res.

[B8] Dopson S, Fitzgerald L (2005). Knowledge to Action: evidence-based healthcare in context.

[B9] Hartley J, Bennington J (2006). Copy and paste, or graft and transplant? Knowledge sharing through inter-organizational networks. Public Money Manage.

[B10] Rashman L, Downe J, Hartley J (2005). Knowledge creation and transfer in the Beacon Scheme: improving services through sharing good practice. Local Government Studies.

[B11] Martin GP, Currie G, Finn R Reconfiguring or reproducing the intra-professional boundaries of expertise? Generalist and specialist knowledge in the modernization of genetics provision in England. International Conference on Organizational Learning, Knowledge and Capabilities; 28 April 2008.

[B12] Haynes P (2003). Managing Complexity in the Public Services.

[B13] Currie G, Waring J, Finn R (2007). The limits of knowledge management for public services modernisation: the case of patient safety and service quality. Public Admin.

[B14] Ferlie E, Fitzgerald L, Wood M, Hawkins C (2005). The nonspread of innovations: the mediating role of professionals. Acad Manage J.

[B15] Currie G, Finn R, Martin G (2008). Accounting for the 'dark side' of new organizational forms: the case of healthcare professionals. Hum Relat.

[B16] Currie G, Suhomlinova O (2006). The impact of institutional forces upon knowledge sharing in the UK NHS: the triumph of professional power and the inconsistency of policy. Public Admin.

[B17] Currie G, Kerrin M (2004). The limits of a technological fix to knowledge management: epistemological, political and cultural issues in the case of intranet implementation. Manage Learn.

[B18] Department of Health (2007). Report of the High Level Group on Clinical Effectiveness.

[B19] Godin G, Belanger-Gravel A, Eccles M, Grimshaw J (2008). Healthcare professionals' intentions and behaviours: a systematic review of studies based on social cognitive theories. Implement Sci.

[B20] Wensing M, Wollersheim H, Grol R (2006). Organizational interventions to implement improvements in patient care: a structured review of reviews. Implement Sci.

[B21] Department of Health (2005). A Guide to Promote a Shared Understanding of the Benefits of Local Managed Networks.

[B22] Transitions Team Department of Health (2005). The Way Forward: the NHS Institute for Learning, Skills and Innovation.

[B23] Research and Development Directorate Department of Health (2006). Best Research for Best Health: a new national health research strategy.

[B24] Pablo AL, Reay T, Dewald JR, Casebeer AL (2007). Identifying, enabling and managing dynamic capabilities in the public sector. J Manage Stud.

[B25] Greenhalgh T, Robert G, Macfarlane F, Bate P, Kyriakidou O (2004). Diffusion of innovations in service organizations: Systematic review and recommendations. Milbank Q.

[B26] Cohen WM, Levinthal DA (1990). Absorptive capacity – a new perspective on learning and innovation. Adm Sci Q.

[B27] Zahra SA, George G (2002). Absorptive capacity: A review, reconceptualization, and extension. Acad Manage Rev.

[B28] Bosch FAJ Van den, van Wijk R, Volberda HW, Easterby-Smith M, Lyles MA (2005). Absorptive capacity: antecedents, models, and outcomes. The Blackwell Handbook of Organizational Learning and Knowledge Management.

[B29] Lane PJ, Koka BR, Pathak S (2006). The reification of absorptive capacity: A critical review and rejuvenation of the construct. Acad Manage Rev.

[B30] Jones O (2006). Developing absorptive capacity in mature organizations: the change agent's role. Manage Learn.

[B31] Todorova G, Durisin B (2007). Absorptive capacity: valuing a reconceptualization. Acad Manage Rev.

[B32] Bosch FAJ Van den, Volberda HW, de Boer M (1999). Coevolution of firm absorptive capacity and knowledge environment: organizational forms and combinative capabilities. Organ Sci.

[B33] Jansen JJ, Van den Bosch FA, Volberda HW (2005). Managing potential and realized absorptive capacity: How do organizational antecedent's matter?. Acad Manage J.

[B34] Fosfuri A, Tribó JA (2008). Exploring the antecedents of potential absorptive capacity and its impact on innovation performance. Omega.

[B35] Easterby-Smith M, Graça M, Antonacopoulous E, Ferdinand J (2008). Absorptive capacity: a process perspective. Manage Learn.

[B36] Scarbrough H, Laurent S, Bresnen M, Edelman L, Newell S, Swan J Learning from projects: the interplay of absorptive and reflective capacity. Organizational Learning and Knowledge 5th International Conference; 20 May 2003.

[B37] Braadbaart O, Yusnandarshah B (2008). Public sector benchmarking: a survey of scientific articles, 1990–2005. Int Rev Adm Sci.

[B38] McColl A, Smith H, White P, Field J (1998). General practitioners' perceptions of the route to evidence based medicine: a questionnaire survey. BMJ.

[B39] Newman K, Pyne T, Leigh S, Rounce K, Cowling A (2000). Personal and organizational competencies requisite for the adoption and implmentation of evidence-based healthcare. Health Serv Manage Res.

[B40] Feasey S, Fox C (2001). Benchmarking evidence-based care. Paediatr Nurs.

[B41] Fanning MF, Oakes DW (2006). A tool for quantifying organizational support for evidence-based practice change. J Nurs Care Qual.

[B42] Wallin L, Ewald U, Wikbad K, Scott-Findlay S, Arnetz BB (2006). Understanding work contextual factors: A short-cut to evidence-based practice?. Worldviews Evid Based Nurs.

[B43] Baker GR, King H, MacDonald JL, Horbar JD (2003). Using organizational assessment surveys for improvement in neonatal intensive care. Pediatrics.

[B44] Weiner BJ, Amick H, Lee SYD (2008). Conceptualization and measurement of organizational readiness for change: a review of the literature in health services research and other fields. Med Care Res Rev.

[B45] Helfrich CD, Li YF, Mohr DC, Meterko M, Sales AE (2007). Assessing an organizational culture instrument based on the Competing Values Framework: exploratory and confirmatory factor analyses. Implement Sci.

[B46] Funk SG, Champagne MT, Wiese RA, Tornquist EM (1991). BARRIERS: the Barriers to Research Utilization Scale. Appl Nurs Res.

[B47] Watson B, Clarke C, Swallow V, Forster S (2005). Exploratory factor analysis of the research and development culture index among qualified nurses. J Clin Nurs.

[B48] Addicott R, McGivern G, Ferlie E (2006). Networks, organizational learning and knowledge management: NHS cancer networks. Public Money Manage.

[B49] Gabbay J, le May A (2004). Evidence based guidelines or collectively constructed "mindlines?" – Ethnographic study of knowledge management in primary care. Br Med J.

[B50] Tucker AL, Nembhard IM, Edmondson AC (2007). Implementing new practices: An empirical study of organizational learning in hospital intensive care units. Manage Sci.

[B51] Berta W, Teare GF, Gilbart E, Ginsburg LS, Lemieux-Charles L, Davis D, Rappolt S (2005). The contingencies of organizational learning in long-term care: factors that affect innovation adoption. Health Care Manage Rev.

[B52] Finn R, Waring J (2006). Organizational barriers to architectural knowledge and teamwork in operating theatres. Public Money Manage.

[B53] Reardon JL, Davidson E (2007). An organizational learning perspective on the assimilation of electronic medical records among small physician practices. Eur J Inform Sys.

[B54] Nutley S, Davies H, Walter I (2004). Learning From Knowledge Management.

[B55] Estabrooks CA, Thompson DS, Lovely JJE, Hofmeyer A (2006). A guide to knowledge translation theory. J Contin Educ Health Prof.

[B56] Mitton C, Adair CE, McKenzie E, Patten SB, Perry BW (2007). Knowledge transfer and exchange: review and synthesis of the literature. Milbank Q.

[B57] Woolf SH (2008). The meaning of translational research and why it matters. JAMA-J Am Med Assoc.

[B58] Lockyer J, Gondocz ST, Thiverge RL (2004). Knowledge translation: the role and place of practice reflection. J Contin Educ Health Prof.

[B59] Ghaye T (2008). Building the Reflective Healthcare Organisation.

[B60] Lyles MA, Easterby-Smith M, Easterby-Smith M, Lyles MA (2005). Organizational learning and knowledge management: agendas for future research. The Blackwell Handbook of Organizational Learning and Knowledge Management.

[B61] Ricceri F (2008). Intellectual Capital and Knowledge Management: strategic management of knowledge resources.

[B62] Lloria MB (2008). A review of the main approaches to knowledge management. Knowledge Management Research & Practice.

[B63] Bali RK, Dwiveci AN (2007). Healthcare knowledge management: issues, advances and successes.

[B64] Easterby-Smith M, Lyles MA, Easterby-Smith M, Lyles MA (2005). Introduction: Watersheds of organizational learning and knowledge management. The Blackwell Handbook of Organizational Learning and Knowledge Management.

[B65] Nonaka I, Takeuchi H (1995). The Knowledge Creating Company: how Japanese companies create the dynamics of innovation.

[B66] Crossan MM, Lane HW, White RE (1999). An organizational learning framework: From intuition to institution. Acad Manage Rev.

[B67] Zollo M, Winter SG (2002). Deliberate learning and the evolution of dynamic capabilities. Organ Sci.

[B68] Boisot MH, Choo CW, Bontis N (2002). The creation and sharing of knowledge. The Strategic Management of Intellectual Capital and Organizational Knowledge.

[B69] Cook SDN, Brown JS (1999). Bridging epistemologies: The generative dance between organizational knowledge and organizational knowing. Organ Sci.

[B70] Orlikowski WJ (2002). Knowing in practice: enacting a collective capability in distributed organizing. Organ Sci.

[B71] Nicolini D, Gherardi S, Yanow D (2003). Knowing in Organizations: a practice-based approach.

[B72] Gherardi S (2006). Organizational Knowledge: the texture of workplace learning.

[B73] Blackler F, Crump N, McDonald S (2000). Organizing processes in complex activity networks. Organization.

[B74] Brown JS, Duguid P (2001). Knowledge and organization: a social-practice perspective. Organ Sci.

[B75] Blackler F, Choo CW, Bontis N (2002). Knowledge, knowledge work, and organizations: an overview and interpretation. The Strategic Management of Intellectual Capital and organizational Knowledge.

[B76] Scarbrough H, Swan J, Easterby-Smith M, Lyles MA (2005). Discourses of knowledge management and the learning organization: their production and consumption. The Blackwell Handbook of Organizational Learning and Knowledge Management.

[B77] Marshall N (2008). Cognitive and practice-based theories of organizational knowledge and learning: incompatible or complementary?. Manage Learn.

[B78] Spender JC (2008). Organizational learning and knowledge management: whence and whither?. Manage Learn.

[B79] Chiva R, Alegre J (2005). Organizational learning and organizational knowledge – toward the integration of two approaches. Manage Learn.

[B80] Bapuji H, Crossan M, Jiang GL, Rouse MJ Organizationallearning: a systematic review of the literature. International Conference on Organizational Learning, Knowledge and Capabilities (OLKC) 2007; 14 June 2007.

[B81] Vera D, Crossan M, Easterby-Smith M, Lyles MA (2005). Organizational learning and knowledge management: towards an integrative framework. The Blackwell Handbook of Organizational Learning and Knowledge Management.

[B82] Nonaka I, Peltokorpi V (2006). Objectivity and subjectivity in knowledge management: a review of 20 top articles. Knowledge and Process Management.

[B83] Kuhn T, Jackson MH (2008). A framework for investigating knowing in organizations. Management Communication Quarterly.

[B84] Sheffield J (2008). Inquiry in health knowledge management. Journal of Knowledge Management.

[B85] Savory C Knowledge translation capability and public-sector innovation processes. International Conference on Organizational Learning, Knowledge and Capabilities (OLKC); 20 March 2006.

[B86] Mulgan G (2007). Ready or Not? Taking innovation in the public sector seriously.

[B87] Foss NJ (2007). The emerging knowledge governance approach: Challenges and characteristics. Organization.

[B88] Martin B, Morton P Organisational conditions for knowledge creation – a critical realist perspective. Annual Conference of the International Association for Critical Realism (IACR); 11 July 2008.

[B89] Kontos PC, Poland BD (2009). Mapping new theoretical and methodological terrain for knowledge translation: contributions from critical realism and the arts. Implement Sci.

[B90] Chiva R, Alegre J, Lapiedra R (2007). Measuring organisational learning capability among the workforce. Int J Manpower.

[B91] van Aken JE (2004). Management research based on the paradigm of the design sciences: the quest for field-tested and grounded technological rules. J Manage Stud.

[B92] Newell S, Robertson S, Scarborough M, Swan J (2002). Making Knowledge Work.

[B93] Canadian Health Services Research Foundation (2005). Is research working for you? A self assessment tool and discussion guide for health services management and policy organizations.

[B94] Templeton GF, Lewis BR, Snyder CA (2002). Development of a measure for the organizational learning construct. J Manage Inform Syst.

[B95] Goh S, Richards G (1997). Benchmarking the learning capability of organizations. European Management Journal.

[B96] Jerez-Gomez P, Cespedes-Lorente J, Valle-Cabrera R (2005). Organizational learning capability: a proposal of measurement. J Bus Res.

[B97] Hooff B van den, Vijvers J, de Ridder J (2003). Foundations and applications of a knowledge management scan. European Management Journal.

[B98] Metcalfe CJ, Hughes C, Perry S, Wright J, Closs J (2000). Research in the NHS: a survey of four therapies. British Journal of Therapy and Rehabilitation.

[B99] Champion VL, Leach A (1986). The relationship of support, availability, and attitude to research utilization. J Nurs Adm.

[B100] Champion VL, Leach A (1989). Variables related to research utilization in nursing: an empirical investigation. J Adv Nurs.

[B101] Alcock D, Carroll G, Goodman M (1990). Staff nurses' perceptions of factors influencing their role in research. Can J Nurs Res.

[B102] Sveiby KE, Simons R (2002). Collaborative climate and effectiveness of knowledge work – an empirical study. Journal of Knowledge Management.

[B103] American Productivity and Quality Center (APQC) (2008). The Knowledge Management Assessment Tool (KMAT).

[B104] Hult GTM, Ferrell OC (1997). Global organizational learning capacity in purchasing: Construct and measurement. J Bus Res.

[B105] Horsley JA, Crane J, Bingle JD (1978). Research utilization as an organizational process. J Nurs Adm.

[B106] Brett JDL (1986). Organizational and Integrative Mechanisms and Adoption of Innovation by Nurses. PhD Thesis.

[B107] Clarke HF (1991). ABC Survey.

[B108] Crane J (1990). Factors Associated with the Use of Research-Based Knowledge in Nursing. PhD Thesis.

[B109] Darroch J (2003). Developing a measure of knowledge management behaviors and practices. Journal of Knowledge Management.

[B110] Lopez SP, Peon JMM, Ordas CJV (2004). Managing knowledge: the link between culture and organizational learning. Journal of Knowledge Management.

[B111] Grimshaw JM, Shirran L, Thomas R, Mowatt G, Fraser C, Bero L, Grilli R, Harvey E, Oxman A, O'Brien MA (2001). Changing provider behavior: an overview of systematic reviews of interventions. Med Care.

[B112] Swan J, McInerney CR, Day RE (2007). Managing knowledge for innovation: production, process and practice. Knowledge Management: from knowledge objects to knowledge processes.

[B113] Gerrish K, Ashworth P, Lacey A, Bailey J, Cooke J, Kendall S, McNeilly E (2007). Factors influencing the development of evidence-based practice: a research tool. J Adv Nurs.

[B114] Kelly DR, Lough M, Rushmer R, Wilkinson JE, Greig G, Davies HTO (2007). Delivering feedback on learning organization characteristics – using a Learning Practice Inventory. J Eval Clin Pract.

[B115] McCormack B, McCarthy G, Wright J, Coffey A, Slater P (2008). Development of the Context Assessment Index (CAI).

[B116] Taylor WA, Wright GH (2004). Organizational readiness for knowledge sharing: challenges for public sector managers. Information Resources Management Journal.

[B117] Matthews J, Shulman AD (2005). Competitive advantage in public-sector organizations: explaining the public good/sustainable competitive advantage paradox. J Bus Res.

