# Supplementary material for: What can management theories offer evidence-based practice? A comparative analysis of measurement tools for organisational context
Source: Implement Sci. 2009 May 19;4:28. doi: 10.1186/1748-5908-4-28 (PMC2694144; doi:10.1186/1748-5908-4-28)
Supplement: Additional file 1 — Measurement tools identified by the search. Titles and bibliographic reference for all measurement tools identified as potentially relevant. [file 1748-5908-4-28-S1.doc]

**Additional file 1: Measurement tools identified by the search**

|  | **Research activity/research utilization** |
| --- | --- |
| **ABC**  **BARR**  **BART**  **KEYS**  **NDF**  **RUS**  **RUSI**  **RUIN**  **R&D** | **ABC Survey [107]**  Attitudes on Nursing Research Scale [118]  Attitudes Towards Nursing Research [119]  **BARRIERS Scale [46]**  **Barriers and Attitudes to Research in Therapies [98]**  Edmonton Research Orientation Scale [120]  Factors Encouraging and Discouraging the Use of Nursing Research Findings [121]  **KEYS - Knowledge Exchanges Yields Success Questionnaire[93]**  **Nursing Department Form [106]**  NPQ - Nursing Practice Questionnaire [122]  Nursing Research Survey [123]  PROBE questionnaire [124]  Research Participation Questionnaire [125]  **Research Utilization Scale [99,100]**  **Research Utilization Survey Instrument [105,108]**  **Research Use in Nursing Practice Instrument [101]**  Research Survey [126]  **R&D Culture Index [47]** |
|  | **Knowledge Management & Learning Organisation** |
| **CCS**  **KMAT**  **KMQ**  **KMS**  **OLC1**  **OLC2**  **OLC3**  **OLS1**  **OLS2** | **Collaborative Climate Survey [102]**  Information Culture Questionnaire [127]  **KMAT Knowledge Management Assessment Tool [103]**  **Knowledge Management Questionnaire [109]**  Knowledge Management Performance Scorecard [128]  **Knowledge Management Scan [97]**  Knowledge Sharing Effectiveness Inventory [129]  **Organisational Learning Capacity [104]**  **Organizational Learning Capability Scale [96]**  **Organizational Learning Construct [94]**  **Organizational Learning Scale [110]**  **Organizational Learning Survey [95]** |
|  | **Research activity/research utilization** |
| **ABC**  **BARR**  **BART**  **KEYS**  **NDF**  **RUS**  **RUSI**  **RUIN**  **R&D** | **ABC Survey [46]**  Attitudes on Nursing Research Scale [67]  Attitudes Towards Nursing Research [68]  **BARRIERS Scale [10]**  **Barriers and Attitudes to Research in Therapies [60]**  Edmonton Research Orientation Scale [69]  Factors Encouraging and Discouraging the Use of Nursing Research Findings [70]  **KEYS - Knowledge Exchanges Yields Success Questionnaire[40]**  **Nursing Department Form [45]**  NPQ - Nursing Practice Questionnaire [71]  Nursing Research Survey [72]  PROBE questionnaire [73]  Research Participation Questionnaire [74]  **Research Utilization Scale [61,62]**  **Research Utilization Survey Instrument [44,47]**  **Research Use in Nursing Practice Instrument [63]**  Research Survey [75]  **R&D Culture Index [11]** |
|  | **Knowledge Management & Learning Organisation** |
| **CCS**  **KMAT**  **KMQ**  **KMS**  **OLC1**  **OLC2**  **OLC3**  **OLS1**  **OLS2** | **Collaborative Climate Survey [64]**  Information Culture Questionnaire [76]  **KMAT Knowledge Management Assessment Tool [65]**  **Knowledge Management Questionnaire [48]**  Knowledge Management Performance Scorecard [77]  **Knowledge Management Scan [59]**  Knowledge Sharing Effectiveness Inventory [78]  **Organisational Learning Capacity [66]**  **Organizational Learning Capability Scale [43]**  **Organizational Learning Construct [41]**  **Organizational Learning Scale [49]**  **Organizational Learning Survey [42]** |

Items in bold font = included for item extraction
