# Supplementary material for: What can management theories offer evidence-based practice? A comparative analysis of measurement tools for organisational context
Source: Implement Sci. 2009 May 19;4:28. doi: 10.1186/1748-5908-4-28 (PMC2694144; doi:10.1186/1748-5908-4-28)
Supplement: Additional file 2 — Details of development and psychometric testing of included measurement tools. Provides background information on psychometric properties of included measurement tools. [file 1748-5908-4-28-S2.doc]

**Additional file 2 : Details of development and psychometric testing of included measurement tools**

| **Tool name, author, date** | **Description + conceptual/theoretical basis** | **Conceptual/**  **theoretical basis** | **Populations tested** | **Development and psychometric testing, sensitivity to change.** |
| --- | --- | --- | --- | --- |
| **ABC Survey[107]** | Individually completed instrument to describe the climate and infrastructure resources for nursing research activities. | Not explicit | Nurses, Canada | Developed from a previous questionnaire. Content validity was tested through peer review. Varcoe & Hilton[118] report the internal consistency range of the subscales as 0.79 to 092. |
| **BARRIERS Scale (BARR)[46]** | Individually completed 28 item scale divided into 4 sub scales which rate the extent to which barriers related to setting, therapist, presentation of findings and qualities of research itself prevent research utilisation by individual practitioners. | Diffusion of Innovation | Nurses and allied health professionals, USA, Europe, Australia, Canada | Developed from research and informal data gathered from nurses. Cronbach’s alpha >0.70. Score range means, SD, Kurtosis and skew indicated well-behaved and normal distributions for each scale. Preliminary estimates of test retest reliability 1 week interval: Pearson’s correlation range 0.68 – 0.83. Extensive replication and revision has been reported. |
| **Barriers and Attitudes to Research in Therapies (BART)[98]** | Individually completed instrument consisting of three scales measuring perceived barriers to research involvement, use of and importance of research, experience of research. | Not explicit | Allied health professionals, UK | Developed from a previous questionnaire. Content validity determined using therapists representing each profession who completed a questionnaire and were interviewed to check for applicability and missing items. Committee reviewed results and made amendments. Factor analysis undertaken. Cronbach’s alpha suggested moderate to high internal consistency (0.51 to 0.85). |
| **Collaborative Climate Survey (CCS)[102]** | Individually completed 20 item instrument measuring collaborative climate, with three subscales for the characteristics of the individual, leadership, culture, and team attitudes to knowledge sharing. | Knowledge management | Private and public sector organisations, USA, Europe, Australia, Asia | Three panels of experts from R&D in University, Government + private sectors were used to brainstorm items. Cronbach’s alpha > 0.80 for all four scales. Survey has been used extensively internationally, comprising >8,000 responses. |
| **Knowledge Exchange Yields Success Questionnaire (KEYS)[93]** | Organisational level self assessment tool to help organisations identify how they gather and use research and where there is potential for improvement. | Not explicit | Health services, Canada | Good face validity. Data is currently being collected from pilot users of the questionnaire for psychometric testing. |
| **Knowledge Management Assessment Tool (KMAT)[103]** | Organisational level 24 item self-assessment tool with five subscales for leadership, technology, culture, measurement and process, to help sub-units of organisations measure and compare how well they manage knowledge | Knowledge management | Commercial sector, USA | A benchmarking tool originally developed by Arthur Anderson Consulting and now used commercially by the American Productivity and Quality Center (APQC). It has been widely used but original information on psychometrics is unavailable as the company no longer exists, and the APQC data is proprietary. |
| **Knowledge Management Questionnaire (KMQ)[109]** | Organisational level instrument with 97 items and three subscales for processes of knowledge acquisition, knowledge dissemination and responsiveness to knowledge, aiming to measure knowledge management behaviours and practices. | Knowledge management | Medium sized commercial companies, New Zealand. | Generated by exploratory research with senior business managers and literature search in knowledge management. Exploratory factor analysis used, with unidimensionality assessed by confirmatory factor analysis. Adjusted goodness of fit measures were acceptable. Convergent, discriminant and predictive validity assessed, |
| **Knowledge Management Scan (KMS)[97]** | Individually completed 75 item instrument with two subscales for knowledge management processes and organisational characteristics, which aimed to measure factors influencing knowledge management. | Knowledge management | Public services and commercial companies, Holland | Developed from knowledge management literature. Testing for item homogeneity and consistency across 3 cases (organisations) is reported, with Cronbach’s alpha above 0.60 for the majority of the scales, except for autonomy and communication climate. Dimensionality tested by factor analysis, with consistency in multidimensional scales. |
| **Nursing Department Form (NDF)[106]** | Organisation level instrument containing 42 questions related to the existence and use of organisational integrative mechanisms for research utilization in nursing. | Diffusion of innovation | Nurses, USA | Developed from a review of the literature and discussions with nurse executives. Cronbach’s alpha total scale coefficient = 0.81. Content validity was assumed due to the literature review and consultation regarding relevance and appropriateness. |
| **Organisational Learning Capacity (OLC1)[104]** | Individually completed 23 item instrument, with four subscales for team, systems, learning and memory orientations, aiming to measure organisational learning capacity. | Organisational learning | Purchasing organization, international | Items based on elements that were well established in the literature, and from 20 case studies. Face and content validity established by expert panel. Construct validity established by factor analysis. Construct reliabilities were >0.70 for all dimensions. Tested for convergent and discriminant validity in one international corporation. |
| **Organisational Learning Capability Scale (OLC2)[96]** | Individually completed 21 item instrument, with five subscales for clarity of mission/purpose, leadership, commitment, experimentation and rewards, teamwork and group problem solving, aiming to measure organisational learning capability. | Organisational learning | Manufacturing firms, Spain | Construct validity assessed by factor analysis. Content validity established by personal interviews. Cronbach’s alpha >0.70. Tested for convergent and discriminant validity in 415 Spanish chemical product manufacturing firms. |
| **Organisational Learning Construct (OLC3)[94]** | Individually completed 28 item instrument, consisting of subscales for attributes of individuals, organisational processes and management, designed to measure engagement in organisational learning. | Organisational learning | Science + research based companies, USA | Items derived from an extensive literature review. Construct validity assessed by factor analysis identified eight dimensions describing organisational learning. Content validity measured by panel of experts. All factors (except organizational grafting) had Cronbach’s alpha statistic greater than 0.5, accepted by authors as sufficient for exploratory research. |
| **Organisational Learning Scale (OLS1)[104]** | Organisational level 26 item self-assessment instrument with four subscales for knowledge distribution, knowledge interpretation, organisational memory and collaborative culture, developed to measure how organisational collaborative culture impacts on organisational learning and performance. | Organisational learning | Large commercial companies, Spain | Developed from exhaustive literature review of the organisational learning literature and previous tools. First, second and third order models developed. All items of the scale had reliability coefficients above 0.06, with all goodness of fit statistics within conservative range, and discriminant validity coefficients between items and factors significant at p=<0.01. The final questionnaire was tested for face validity with experts in knowledge management and organisational learning. |
| **Organisational Learning Survey (OLS2)[95]** | The aim of tool development was to provide information to help managers focus on interventions required to improve learning. | Organisational learning | Public + private sector, Europe | Based on a review of the literature. Construct validity assessed by factor analysis. Content validity tested via focus groups. Cronbach’s alpha of 0.9. Test-retest reliability: r=0.77. Scale shown to have convergent and discriminant validity. Tests in five different organisations suggested it was able to discriminate on characteristics important to learning. |
| **R&D Culture Index (R&D)[47]** | Individually completed18 item instrument intended to assess the strength of organisational R&D culture | Not explicit | Nurses, UK | Developed from findings of qualitative research with NHS clinical nurses and managers. Content validity established by expert review and construct validity by factor analysis. Internal consistency – coefficient alpha 0.86. |
| **Research Use in Nursing Practice Instrument (RUIN)[101]** | Individually completed instrument measuring factors influencing general research use in nursing. Six sections including research values, roles, interest, and experience, and eleven statements about perception of organisational research climate. | Not explicit | Nurses, Canada | Developed from a search of the literature. Items were reviewed for face validity by clinical experts and nurse researchers. Reliability analyses of composite scores alpha range 0.71 to 0.87. Robichaud-Ekstrand and Sherrard (1994) performed more detailed reliability analysis, with a reliability coefficient for perception of research climate items of 0.72. |
| **Research Utilization Scale (RUS)[99,100]** | Individually completed instrument divided into three subscales describing attitude, availability, and support for general use of research by nurses. Seven items for support. | Not explicit | Nurses + Occupational Therapists in USA, Canada, Europe, UK | Developed from past research. Cronbach’s alpha >0.70, with some assessment of content validity by professional opinion and self-report validity assessed by follow up interview. Several studies have revised and used the scale |
| **Research Utilization Survey Instrument (RUSI)[105,108]** | Individually completed 360 item instrument measuring characteristics of knowledge, channel of communication, individual and organisational knowledge-related factors associated with specific research utilization by nurses. | Knowledge utilization, diffusion of innovation, change management | Nurses, USA | Developed from a previous large scale research project. Cronbach’s alpha > 0.7 Construct validity was determined by predicting associations, with minimal levels of acceptability set. |
